# Co-Encapsulating the Fusogenic Peptide INF7 and Molecular Imaging Probes in Liposomes Increases Intracellular Signal and Probe Retention

**DOI:** 10.1371/journal.pone.0120982

**Published:** 2015-03-27

**Authors:** Scott R. Burks, Eric A. Legenzov, Erik W. Martin, Changqing Li, Wuyuan Lu, Joseph P. Y. Kao

**Affiliations:** 1 Center for Biomedical Engineering and Technology and Department of Physiology, University of Maryland, Baltimore, Maryland, 21201, United States of America; 2 Institute of Human Virology and Department of Biochemistry and Molecular Biology, University of Maryland, Baltimore, Maryland, 21201, United States of America; University of California, Berkeley, UNITED STATES

## Abstract

Liposomes are promising vehicles to deliver diagnostic and therapeutic agents to cells *in vivo*. After uptake into cells by endocytosis, liposomes are degraded in the endolysosomal system. Consequently, the encapsulated cargo molecules frequently remain sequestered in endosomal compartments; this limits their usefulness in many applications (e.g. gene delivery). To overcome this, various fusogenic peptides have been developed to facilitate delivery of liposomally-encapsulated molecules into the cytosol. One such peptide is the pH-sensitive influenza-derived peptide INF7. Liposomal delivery of imaging agents is an attractive approach for enabling cell imaging and cell tracking *in vivo*, but can be hampered by inadequate intracellular accumulation and retention of probes caused by exocytosis (and possible degradation) of endosome-entrapped probes. Such signal loss could be minimized by facilitating escape of probe molecules from endolysosomal compartments into the cytosol. We investigated the ability of co-encapsulated INF7 to release liposomally-delivered rhodamine fluorophores into the cytosol after endosomal acidification/maturation. We co-encapsulated INF7 and fluorescent rhodamine derivatives having vastly different transport properties to show that after endocytosis by CV1 cells, the INF7 peptide is activated by acidic endosomal pH and facilitates efficient release of the fluorescent tracers into the cytosol. Furthermore, we show that INF7-facilitated escape from endosomes markedly enhanced retention of tracers that cannot be actively extruded from the cytosol. Minimizing loss of intracellular probes improves cellular imaging by increasing the signal-to-noise ratio of images and lengthening the time window that imaging can be performed. In particular, this will enhance *in vivo* electron paramagnetic resonance imaging, an emergent magnetic resonance imaging modality requires exogenous paramagnetic imaging agents and is highly promising for cellular and molecular imaging.

## Introduction

**Fig 1:**
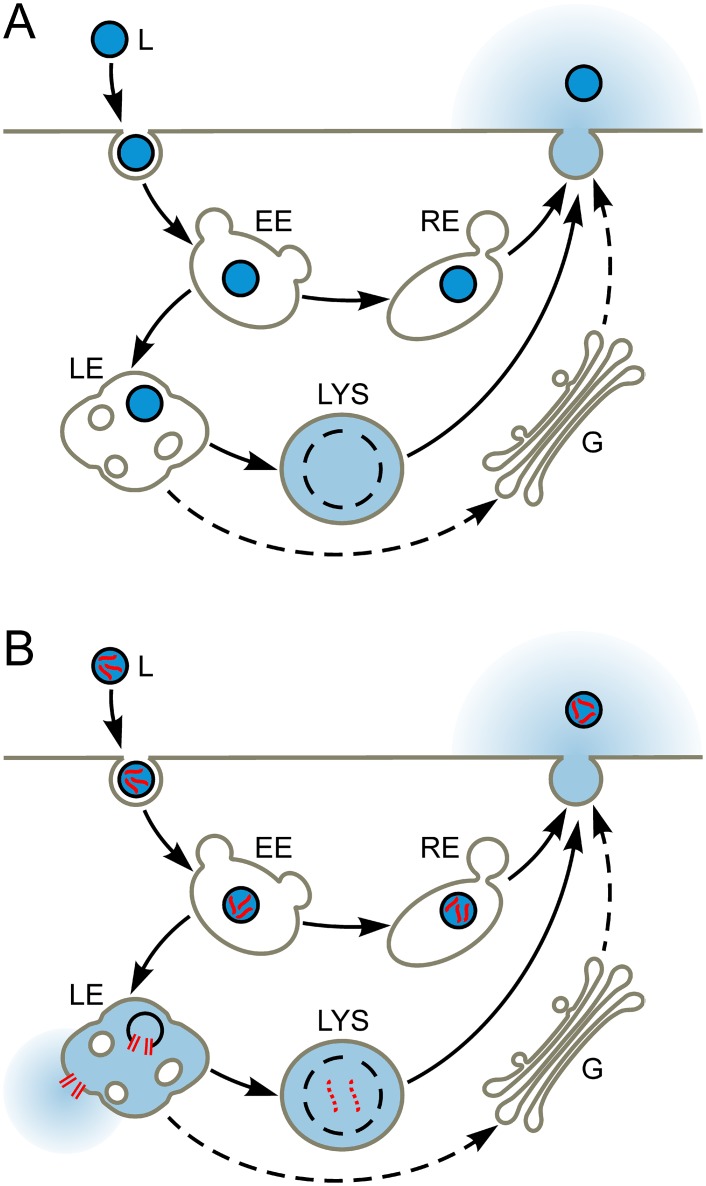
Liposome endocytosis and the endo-lysosomal pathway. **A.** A liposome (L; dark blue lumenal content) is endocytosed and enters an early endosome (EE). Thereafter, it can pass through the recycling endosome (RE) and be exocytosed, or pass through the late endosome (LE) and enter the lysosome (LYS), where it is degraded and releases its lumenal contents. Potential trafficking through the Golgi (G) is also shown. **B.** Liposome whose lumenal content includes the pH-sensitive fusogenic peptide INF7 (red squiggles) is endocytosed. The acidic pH of the late endosome activates the INF7 to permeabilize the liposomal and endosomal membranes and thus facilitate release of liposomal contents into the cytosol.

*In vivo* cellular imaging is an invaluable clinical and biomedical research tool that permits localization, tracking, and physiological monitoring of specific cells and tissues. The utility and versatility of an imaging modality is often enhanced by exogenous imaging probes or contrast agents. Indeed, such agents are obligatory for some imaging modalities. *In vivo* cellular imaging requires strategies to deliver imaging probes selectively, and in sufficient quantities, to cells of interest. Liposomes are attractive carriers for many therapeutic and diagnostic agents, including imaging probes. This is due to their biocompatibility [1], controllable pharmacokinetic properties [2, 3], and ability to target specific cell types, including tumors [4–6]. Liposomes have been used to label cells with imaging agents for nearly all biomedical imaging modalities [7–9] and can be used both *in vitro* and *in vivo* [6, 9]. Endocytosis is the principal mode of liposome uptake by cells. Susceptibility of liposomes to endocytosis can be modulated by chemical modification of the liposome—e.g., by modifying the lipid composition or surface charge [10, 11], and by decorating the liposome surface with specific polymers [2, 12, 13], ligands [14–16] or antibodies [5, 6, 17]. After endocytosis, liposomes are degraded in the endolysosomal pathway (Fig. 1A), and the material encapsulated in the liposome lumen is released into the endolysosomal compartment [18, 19]. Lumenal components that are large hydrophilic molecules or molecules bearing multiple ionic charges cannot readily cross biomembranes and thus remain entrapped in endolysosomal compartment. This is inconsequential for cellular imaging applications that only require imaging probes to be localized intracellularly. However, endosomal retention creates obstacles that limit the full potential of cellular imaging. First, new developments in cellular imaging aim to probe intracellular physiology *in vivo*, which is not possible if probes are confined to endosomes and isolated from other physiologically-relevant compartments. Second, intracellular signal retention of endosome-entrapped probes can be poor. This can lower the signal-to-noise ratio (SNR) and shorten the time window for cellular imaging. Imaging probes trafficked through the endosomal system can be shuttled into secretory pathways and eventually exocytosed (Fig. 1A) [20, 21]. Imaging probes in the endolysosomal pathway also could be destroyed through cellular degradative mechanisms. Both of these scenarios would lead to reduction of imageable probe signal. Therefore, strategies to facilitate escape of liposomally-encapsulated imaging probes from the endolysosomal pathway into the cytosol would improve many cellular imaging applications.

Endosomal escape after endocytosis is critical for delivery of agents such as nucleic acids, which require access to the nucleo-cytosolic compartment. Nucleic acids are highly susceptible to endosomal/lysosomal degradation and are generally ineffective when they are excluded from the nucleo-cytosolic compartment. A variety of fusogenic peptides (FP) have been engineered to enable endosomal escape (for review see [22]). Many FPs are virally-derived sequences that evolved to enable virion entry into the cytosol post-infection. The influenza hemagglutinin membrane protein HA2 is a pH-sensitive fusogenic protein that is activated by endosomal acidification to allow escape of the viral genome into the cytosol [23, 24]. The INF7 peptide is a glutamine-enriched analogue of the membrane-disruptive segment of HA2 (amino acids 1–23) that exhibits greater pH sensitivity and has been shown to preserve the integrity of membrane bilayers at pH > 6, but disrupt them at endosomal pH (~5.0) [25, 26]. Therefore, INF7 represents an ideal FP to investigate endosomal escape of imaging probes. Previous demonstrations of INF7-mediated endosomal escape used nucleic acids encoding reporter proteins or the potent endotoxin, diphtheria toxin [25, 27] Such studies may over-estimate the efficacy of INF because even a few copies of reporter-encoding nucleic acids escaping into the nucleo-cytosolic compartment can result in detectable reporter expression and even a single molecule of diphtheria toxin entering the cytosol can kill a cell [28]. Therefore, it remains unclear whether INF7 can promote escape of large quantities of imaging probes into the cytosol. It is also unknown whether intracellular probe retention would be improved by having probe molecules reside in the cytosol rather than the endo-lysosomal pathway.

**Fig 2:**
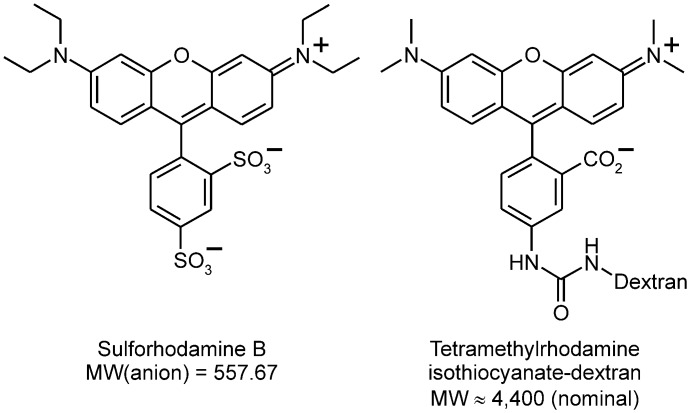
Fluid-phase fluorescent tracers.

We demonstrate in this study that INF7 can be co-encapsulated in liposomes with high concentrations of rhodamine-type fluorophores (>10 mM). At such concentrations, rhodamine fluorescence is quenched, making intact un-endocytosed liposomes appear “dark”. After endocytosis by CV1 cells (African green monkey kidney epithelial cell line), liposomes are disrupted in the endo-lysosomal compartment [18]. This dilutes encapsulated fluorophores into the much-larger endo-lysosomal volume, whereupon de-quenching occurs and robust endo-lysosomal fluorescence is generated [9]. INF7 is activated by endosomal acidification and should efficiently release fluorophores into the cytosol (Fig. 1B). We demonstrate endosomal escape by encapsulating sulforhodamine B (SR) and rhodamine-conjugated dextran (RD; MW ~4,400) (Fig. 2), which are both retained by endosomes following endocytosis, but have very different extrusion rates [29, 30] once they have escaped from the endosome into the cytosol. SR is a small molecule bearing three ionic charges at physiologic pH and is rapidly extruded from cells by cytosolic transport mechanisms [29]. Thus when SR is released from endosomes into the cytosol by INF7, its intracellular retention time is actually shorter than if it remained in the endolysosomal pathway. Like all dyes conjugated to dextran polymers, RD is not extruded from the cytosol. Thus when RD is released into the cytosol by INF7, its intracellular retention time is longer than if it remained in the endolysosomal pathway. These findings are especially relevant to electron paramagnetic resonance imaging (EPRI). EPRI is an emerging magnetic resonance modality that uses exogenous paramagnetic molecular probes such as nitroxides. EPRI with tailored nitroxides can be employed for deep tissue imaging to identify and track specific cell populations and image cellular physiology *in vivo* [17]. EPRI of cells labeled by nitroxides delivered through targeted liposomes is promising, but cell labeling currently suffers from poor retention of nitroxide signal. Methodology to facilitate endosomal escape, coupled with improved design of nitroxide molecules for extended intracellular retention, should advance cellular and physiological imaging by EPRI.

## Materials and Methods

### Peptide Synthesis

INF7 peptide (H_2_N-GLFEAIEGFIENGWEGMIDGWYGC-CO_2_H) was synthesized on an Applied Biosystems 433 A synthesizer using the published DIEA *in situ* neutralization/HBTU activation protocol for Boc solid-phase peptide synthesis (DIEA = *N*,*N*-diisopropylethylamine, HBTU = 2-(1*H*-benzotriazole1yl)-1,1,3,3-tetramethyluronium hexafluorophosphate, Boc = *tert*-butoxycarbonyl) [31, 32]. Specifically, Boc-amino acids (2.2 mmol) were activated in *N*,*N*-dimethylformamide (DMF) for 3 min by 2.0 mmol of HBTU in the presence of 20% DIEA (v/v), and coupled in stepwise fashion for 10 min on 0.25 mmol of PAM resin (PAM = 4-hydroxymethylphenylacetamidomethyl). Trifluoroacetic acid (TFA, 100%) was used for removal of *N*-Boc groups; DMF and CH_2_Cl_2_ were used for batch washes throughout the entire synthesis. The following side chain protections were used: Asn(Xanthyl), Asp(OcHxl), Cys(4-MeBzl), Glu(OcHxl), Tyr(BrZ), Trp(CHO) (cHxl = cyclohexyl, MeBzl = 4-methylbenzyl, BrZ = 2-bromobenzyloxycarbonyl). After chain assembly, the peptides were deprotected and cleaved by anhydrous HF in the presence of 5% *p*-cresol at 0°C for 1 h, followed by precipitation with cold ether.

INF7 monomers were dimerized by oxidative disulfide formation between C-terminal cysteines. The desired product was verified by mass spectrometry and purified by preparative reversed phase (RP) HPLC and subsequently lyophilized. Preparative RP-HPLC was carried out on a Waters Delta Prep 600 system (Milford, MA, USA) equipped with a Vydac preparative C18 column (15–20 μm, 50 × 250 mm). The binary solvent system comprised water containing 0.1% v/v TFA, and acetonitrile containing 0.1% v/v TFA. The desired product eluted as the major HPLC peak and corresponding fractions were collected and lyophilized to a white powder, which gave a unique molecular mass of 5386.5 ± 0.5 Da by electrospray mass spectrometry, in agreement, within experimental error, with the theoretical value of 5386.0 Da calculated on the basis of the average isotopic compositions of dimeric INF7.

The disulfide-linked dimer of INF7 is used in all biological studies presented here. For simplicity, hereafter the term “INF7” refers to the dimer.

### Liposome Preparation and Stability Studies

#### Liposome preparation

Liposomes comprised 1,2-distearoylphosphatidylcholine (DSPC), cholesterol (Chol), L-α-phosphatidylserine (PS; porcine brain isolate), and 1,2-dioleoyl-*sn*-glycero-3-phosphatidylethanolamine-*N*-(carboxyfluorescein) ammonium salt (CF-PE) in the molar ratio 3:2:0.3:0.003 (DSPC:Chol:PS:CF-PE). All lipids were obtained from Avanti Polar Lipids (Alabaster, AL). CF-PE serves as a lipid-phase fluorescent tracer. A solution of 10 μmol phospholipid in 100 μL EtOH was injected into 1 mL of rapidly stirred aqueous solution to be encapsulated (see below). The mixture was extruded 11 times through a 100-nm porosity filter membrane (Nucleopore Track-etch Membrane, Whatman, a division of GE Healthcare, Piscataway, NJ) in a Mini Extruder (Avanti Polar Lipids) to yield a suspension of liposomes. All solutions and the extruder were maintained at >55°C to ensure fluidity of the lipid phase. Encapsulated solutions contained either 90 mM sulforhodamine B (SR; Exciton, Dayton, OH) or 10 mM tetramethylrhodamine isothiocyanate-dextran (RD; MW ~4,000; Sigma-Aldrich, St. Louis, MO) as the fluid-phase fluorescent tracer, with or without the addition of 500 μg/mL INF7 (93 μM dimer concentration). Liposomes were purified on Sephadex G-50 resin (~4 g; GE Healthcare) packed into a FlexColumn (15 × 300 mm; Kimbal-Kontes, Vineland, NJ), and equilibrated with Dulbecco’s phosphate-buffered saline (DPBS). Gel filtration yielded ~4 ml of purified liposome suspension in DPBS (final phospholipid concentration ~2 μmol/mL), which was stored at 4°C until use. We have previously shown that liposomes produced as described above are very stable [33]. INF7 peptide was engineered to be fusogenic at pH ~5 and to have minimal activity at pH >6 [25, 26]. In our experience, liposomes encapsulating INF7 are stable for at least 4 weeks when stored in DPBS at 4°C (see data below), but are always used within 2 weeks of preparation.

For use in fluid-phase endocytosis experiments, liposomes were prepared as described above without incorporating fluorescent tracers; the liposomes were prepared and extruded in DPBS. This preparation is referred to as “empty liposomes”.

#### Stability of liposomes encapsulating INF7 peptide

Stability of liposomes encapsulating INF7 peptide was assayed as a function of 1) pH, and 2) time. A suspension of liposomes encapsulating 90 mM SR and 500 μg/mL INF7 was prepared as described above and was stored at 4°C. For measurements over a wide pH range, an assay buffer of DPBS supplemented with 10 mM citric acid was adjusted with concentrated NaOH (~10 M) to prepared 9 solutions with the following pH values: 3.5, 4.5, 5.0, 5.5, 6.0, 6.5, 7.0, 7.5, 8.45. Measurement confirmed that the pH values of the assay buffers at room temperature (22°C) and 37°C were essentially identical (± 0.02 pH unit); this is consistent with the known insensitivity of citrate and phosphate buffers to temperature. An initial-rate experiment was used to assess liposome stability as a function of pH. Assay buffer (2.5 mL) at a known pH was stirred and thermally equlibrated in a fluorescence cuvette thermostatted at 37°C. Liposome suspension (11.25 μL) was added to the stirred assay solution as the SR fluorescence emission was monitored. Because SR was encapsulated at self-quenching concentration, any loss of liposome integrity would cause SR to leak and be diluted into the bulk assay buffer. The consequent increase in SR fluorescence (de-quenching) is a sensitive read-out of leakage from liposomes. Separate experiments showed that 60 sec of stirring was required to achieve homogeneity in the cuvette; therefore data from the first 65 sec after liposome addition were not used in the analysis. Intensity-vs-time data for the next 500–600 sec were analyzed by nonlinear least-squares, single-exponential curve fitting. The slope (rate) of the fitted curve at 180 sec after liposome addition was used for subsequent analysis. Replicate measurements at each pH were averaged and all average rates were normalized to the slowest rate (which was observed at the highest pH, 8.45). The normalized data were fit to a dose-response function by nonlinear least-squares curve-fitting (S1 Fig.). The midpoint of the fitted dose-response function occurs at pH = 5.64 ± 0.09, consistent with previous observation [25].

In order to assess the stability of the liposomes over time, the de-quenching of SR upon liposome lysis was measured immediately after preparation, and again 4 weeks later. To 2.5 mL of assay buffer (pH 7.5) stirred in a fluorescence cuvette at 22°C was added an 11.25-μL aliquot of liposome suspension, and the SR fluorescence intensity was monitored. After 180 sec, 60 μL of 50% (wt/wt) Triton X-100 was added to lyse the liposomes and release all the SR. Measurement was terminated when the fluorescence had stabilized at its maximum post-lysis value. The average post-lysis (de-quenched) intensity was ratioed against the pre-lysis (quenched) intensity. The ratio determined immediately after liposome preparation was 34.8 ± 6.5 (n = 4); the ratio determined 4 weeks later was 31.0 ± 1.4 (n = 2); the ratios are not significantly different (*p* = 0.174). Thus, the INF7 liposomes stably retain their encapsulated content for at least 4 weeks when stored at 4°C.

### Cell Culture

CV1 cells (ATCC, Manassas, VA) were maintained at 37°C under a 5% CO_2_ atmosphere in Dulbecco’s modified Eagle medium (DMEM) supplemented with 10% (v/v) fetal bovine serum (FBS), 2 mM L-glutamine, 100 U/mL penicillin, and 100 μg/mL streptomycin.

### Cellular uptake of liposomes and unencapsulated fluorophores

#### Cellular uptake of liposomes for microscopic analyses

CV1 cells (~8×10^4^) were plated on 25-mm round No. 1 glass coverslips for 24–48 h. Liposomes encapsulating either SR or RD with and without INF7 were prepared and diluted to a concentration of 0.1 μmol of phospholipid/mL in Hanks’ balanced salt solution (HBSS). CV1 cells were incubated with liposomes for 30 min at 37°C and then washed thrice with divalent-cation-free HBSS (containing no Ca^2+^ or Mg^2+^ but 1 mM ethylenediaminetetraacetic acid disodium salt, Na_2_H_2_EDTA). Thereafter, the cells were maintained in normal (Ca^2+^- and Mg^2+^-containing) HBSS for fluorescence microscopy. Rhodamine fluorescence in cells was imaged before and after the addition of 1% (v/v) acetic acid to the extracellular solution. To examine INF7-mediated release of rhodamine after physiological endosomal acidification, cells were incubated with liposomes at 37°C for 1 hr and washed as described above. Cells were then incubated in normal HBSS at 37°C for an additional 2 hr before imaging.

#### Cellular uptake of fluorophores through fluid-phase endocytosis

CV1 cells were plated at ~35% confluence in replicate 60-mm diameter Petri dishes and allowed to grow for 2 d. For studies on recycling of fluid–phase tracers, the culture medium was removed by aspiration and each dish received 2 mL DMEM (10% FBS), 300 μM SR, and “empty liposomes” at a concentration of 0.12 μmol/mL phospholipid. The dishes were incubated at 37°C for 1 hr. Thereafter, the incubation medium was removed by aspiration, and each dish was washed 3 time with 2 mL DMEM (10% FBS) and once with 2 mL HBSS. Care was taken to ensure that no residual SR-containing medium adhered to the walls of the dishes. After receiving 1.5 mL HBSS each, the dishes were incubated at 37°C. Sets of 3 replicate dishes were removed at 0, 30, 60, 105, 150 and 300 minutes. The HBSS from each dish was collected individually. Each dish then received 1 mL divalent-cation-free DPBS containing 1% w/v Triton X-100 and 2 30-sec episodes of sonication (model G112SP1G, Laboratory Supplies Co., Hicksville, NY) separated by 2 min. Any remaining cells or cell debris were detached with a cell scraper and the total cell lysate from each dish was collected. The HBSS and lysate samples were centrifuged at 20,937 × *g*. SR fluorescence in each sample was quantitated by fluorescence spectroscopy (_exc_ = 564 nm, λ_em_ = 582 nm, determined for the present lysate composition). The relative amounts of intracellular and extracellular SR were computed for each time point.

To assess whether SR could be degraded intracellularly in the several-hour time frame of endocytosis experiments, replicate dishes of cells were loaded with SR and incubated in HBSS exactly as described above. Sets of 3 dishes were removed at 0, 150, and 300 min, and the HBSS in each dish was made up to 1% w/v Triton X-100, 2 mM Na_4_EGTA. The dishes were sonicated and scraped as described above and the total lysate from each dish was collected and centrifuged at 20,937 × *g*. SR fluorescence in each lysate was quantitated fluorometrically (λ_exc_ = 564 nm, λ_em_ = 582 nm).

#### Cellular uptake of liposomes for fluorescence spectroscopy measurements

Replicate 60-mm plastic Petri dishes of CV1 cells were incubated at 37°C with a suspension of liposomes encapsulating SR or RD with and without INF7. Cells were incubated for 4 hr with liposomes at a concentration of 0.1 μmol of phospholipid/mL HBSS. At each time point, cells were washed with HBSS and then released from the plate with trypsin–EDTA solution. Cells were centrifuged at 145×*g* for 3 min, and the pellet was washed twice with divalent-cation-free HBSS. After the final wash, the pellet was resuspended in 1 mL normal HBSS. Cells were lysed with 120 μM digitonin and sonicated for 1 min. SR or RD and CF-PE in the lysates were analyzed by fluorescence spectroscopy. To determine any effects of INF7 on the uptake of liposomes, cells were incubated with liposomes for 30 min and processed as describe above. In each sample, CF-PE fluorescence was first measured; thereafter ethidium bromide (EthBr, 10 μM) was added and EthBr fluorescence was measured.

### Fluorescence Microscopy

Fluorescence images were acquired on an inverted epifluorescence microscope (Eclipse TE200; Nikon Corp., Tokyo, Japan) equipped with a 40× oil-immersion objective (Super Fluor, NA 1.4; Nikon). The excitation light source was a xenon arc lamp coupled to a monochromator (PolyChrome II; TILL Photonics, Gräfelfing, Germany). Fluorescence was passed through an appropriate band-pass filter before capture by a CCD camera (CoolSnap HQ; Roper Scientific, Tucson, AZ). MetaFluor software (Molecular Devices, Downingtown, PA) was used for instrument control and image acquisition.

### Fluorescence Spectroscopy

Fluorescence spectra were recorded on a dual-excitation spectrofluorometer (CM1T-10I; SPEX Industries, Metuchen, NJ); the temperature of the cuvette holder was controlled by a thermostatic water bath/circulator and was monitored with a type-T thermocouple. Instrument control and data acquisition were performed with Datamax software (Galactic Industries Corporation, Salem, NH). Emission and excitation spectra were acquired to determine optimal wavelength settings (CF-PE: λ_ex_ = 490 nm, λ_em_ = 515 nm; Rhodamine (SR and RD): λ_ex_ = 550 nm, λ_em_ = 588 nm; EthBr: λ_ex_ = 310 nm, λ_em_ = 610 nm).

### Image and Statistical Analyses

The mean and SD of pixel intensity in fluorescence images was determined using ImageJ (National Institutes of Health, Bethesda, MD). One-way ANOVAs with post-hoc tests (Scheffe method of means comparison) were used for data sets containing more than two groups. Student’s *t*-test was used for pairwise comparisons. A 95% confidence interval (*p* < 0.05) was used to determine significance. Values are presented as mean ± SD.

## Results

### Retention of SR in the endo-lysosomal pathway

**Fig 3:**
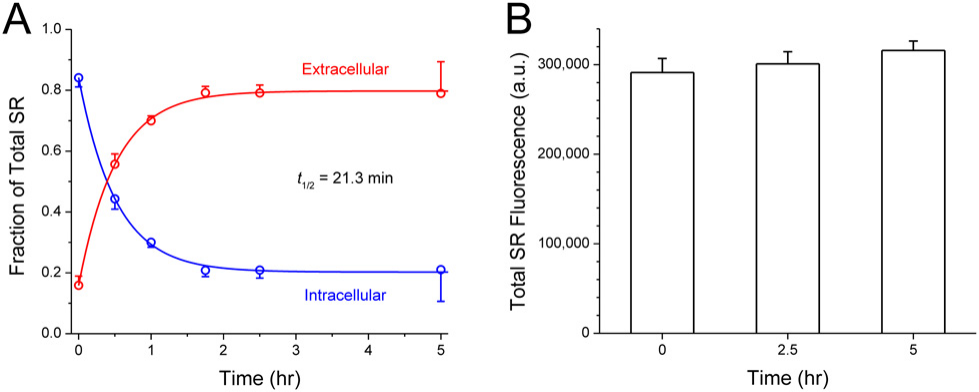
Intracelluar retention of sulforhodamine (SR) tracer after fluid-phase endocytosis. **A.** Dishes of CV1 cells were incubated with 300 μM SR and empty DSPC:PS liposomes for 1 hr to promote fluid-phase endocytosis, and then maintained in HBSS. The proportion of intracellular and extracellular SR was assayed at various times (see Methods for details). The majority (80%) of endocytosed SR reappeared in the extracellular medium with a half-life of *t*_1/2_ = 21.3 ± 1.0 min, while 20% was retained more stably. Solid curves are least-squares single-exponential fits to the data, with exponential time constant τ = 30.8 ± 1.5 min (*t*_1/2_ = τ × ln2 = 0.693·τ). Each data point is the average of 3 replicates. **B.** CV1 cells endocytosed SR as above. At 3 time points thereafter, cells in each dish were lysed and the total amount of SR was assayed by fluorescence spectroscopy. The total SR fluorescence recovered did not differ significantly between time points; each time point is the average of 3 replicates. The means are not significantly different (one-way ANOVA, *p* = 0.159).

We first investigated the intracellular retention time of fluid-phase tracers in the endo-lysosomal pathway. We therefore loaded SR into CV1 cells through fluid-phase endocytosis. Because CV1 cells avidly endocytose liposomes displaying negatively charged lipid headgroups [34], we added empty DSPC:PS liposomes into the SR-containing medium to promote endocytosis. After 1 hr, the cells were washed and transferred into HBSS and the proportion of intracellular and extracellular SR was assessed at various times over a 5-hr period. The results are shown in Fig. 3A: The majority (80%) of fluid-phase tracers taken up by endocytosis into the endolysosomal pathway reappear in the extracellular solution with a half-life of 21.3 min. Interestingly, a minor fraction (20%) of the tracers appear to be quite stably retained, at least on the 5-hr time scale of this experiment. The well-retained fraction could represent tracers that trafficked to stable compartments such as the lysosome and Golgi. In a second, related experiment, we examined if the fluid-phase tracer was degraded over a 5-hour time span. CV1 cells were allowed to take up SR by fluid-phase endocytosis exactly as above. Thereafter, at 0, 2.5 and 5 hr, the *total* amount of SR (extracellular and intracellular) was assayed (*n* = 3 for each time point). As shown in Fig. 3B, the total SR did not diminish with time (*p* = 0.159), which indicates that the SR tracer molecules were not destroyed by cellular mechanisms.

### Activation of INF7 by acetic acid to promote SR release into the cytosol

**Fig 4:**
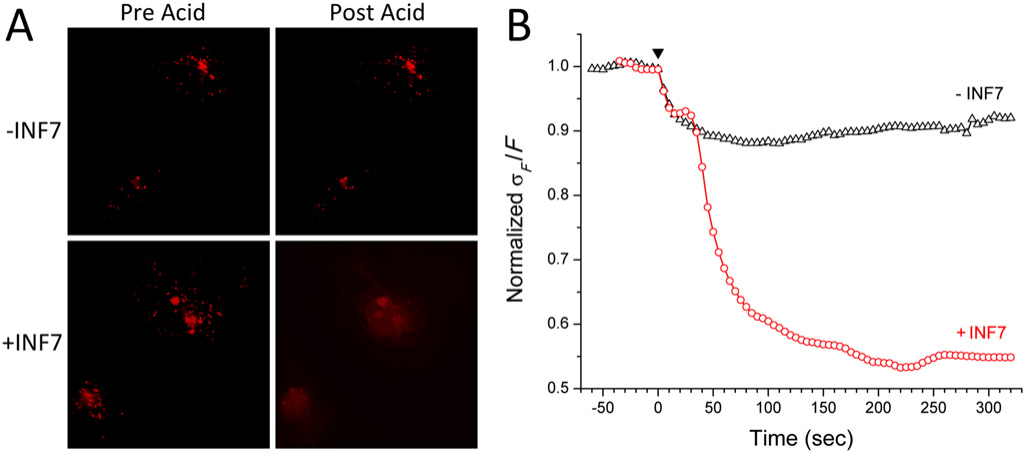
Activating endocytosed INF7 with acetic acid causes endosomal escape of SR into the cytosol. **A.** Liposomes containing SR with or without INF7 were incubated with CV1 cells for 1 hr, washed with Ca^2+^/Mg^2+^-free HBSS containing 1 mM EDTA, and imaged by fluorescence microscopy (Pre Acid); endosomal SR appears as fluorescent puncta. After the addition of 1% acetic acid to the extracellular solution, fluorescence in cells incubated with INF7-containing liposomes (+INF7) becomes cytosolic and more homogeneous. In contrast, in cells incubated with liposomes lacking INF7 (-INF7), the appearance of SR fluorescence is unchanged by acidification. **B.** Time course showing changes in SD of pixel intensity relative to mean intensity (σ*/F*, normalized to mean baseline value before acidification at time 0). Time of addition of acetic acid is indicated by the black arrowhead. Activated INF7 rapidly releases SR into the cytosol, and the more homogeneous distribution of SR leads to lower SD values than in the case of incubation without INF7, where SR remains confined to endosomal compartments. Also see S1 and S2 Videos in Supporting Information.

To investigate endosomal release of fluorophores by INF7, CV1 cells were incubated with liposomes bearing a negative surface charge and encapsulating SR at 90 mM, at which concentration SR fluorescence is quenched. CV1 cells avidly endocytose negatively-charged liposomes [34]; subsequent degradation of liposomes releases SR into the endosomal compartment and the consequent dilution de-quenches SR to restore its fluorescence. Since SR bears three ionic charges at physiological pH it is expected to remain trapped inside endosomes in the absence of INF7. Liposomes, with or without INF7, were incubated with cells for 1 hr. Thereafter, the cells were washed in divalent-cation-free HBSS to remove un-endocytosed liposomes, returned to normal HBSS, and then immediately imaged for SR fluorescence (Fig. 4A). After 1 hour, sufficient liposomal degradation had occurred in the endocytic pathway and SR fluorescence was visualized as intense puncta in both groups. The cells were then rapidly acidified by addition of acetic acid (1% v/v) to the extracellular solution, which attained pH ~3.5; SR in cells incubated with INF7-containing liposomes rapidly dispersed through the intracellular volume and was visualized as more homogenous fluorescence throughout the volume of the cell. Cells that were incubated with liposomes lacking INF7 showed no change in appearance after acidification—demonstrating that without INF7 to facilitate its release, SR remained inside endosomes (S1 Video and S2 Video). The visually apparent changes in intracellular localization of SR after acidification can also be quantitatively analyzed by measuring changes in the standard deviation of pixel intensity ratioed to the mean pixel intensity (σ_*F*_ */F*; Fig. 4B). Before acidification, endosomal localization of SR is seen as highly spatially heterogeneous distribution of fluorescence in the cells, and σ_*F*_ */F* is high for both groups of cells. In the cells incubated with INF7-containing liposomes, after acidification and consequent INF7 activation, SR fluorescence becomes more homogenously distributed in the cells, resulting in lower σ_*F*_ */F* values. In contrast, cells incubated with liposomes containing no INF7, SR fluorescence remains spatially heterogeneous, and the σ_*F*_ */F* value remains high.

### Intracellular distribution and retention of SR during endosomal maturation

**Fig 5:**
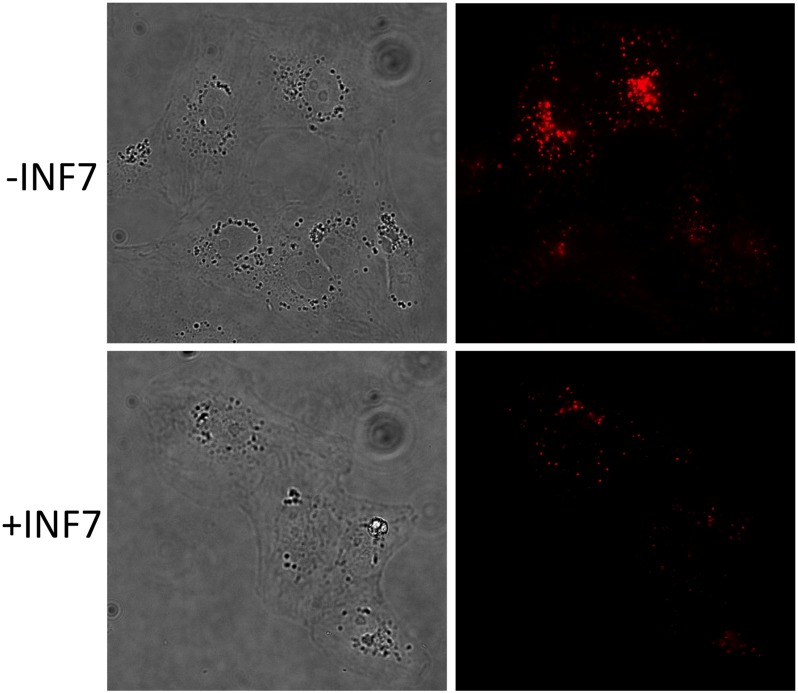
INF7 mediates release of endosomal SR into the cytosol. Liposomes containing SR with or without INF7 were incubated with CV1 cells for 1 hr, washed to remove un-endocytosed liposomes, and allowed to incubate without liposomes for an additional 2 hr. SR remains confined in endosomes without INF7 (-INF7), but after release into the cytosol by INF7, SR is rapidly transported from the cytosol into the extracellular volume (+INF7) leading to much dimmer fluorescence in cells incubated INF7-containing liposomes. See main text for more detailed mechanistic explanation.

**Fig 6:**
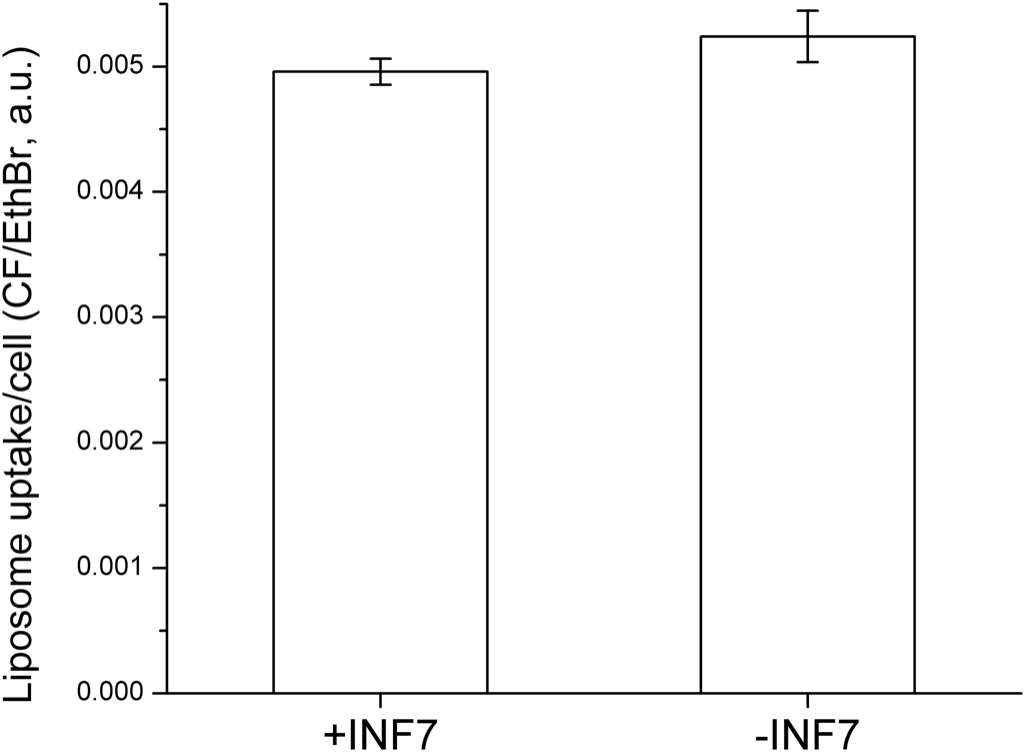
Liposomal encapsulation of INF7 does not alter the quantity of liposomes endocytosed by CV1 cells. Liposomes containing CF-PE as a lipid marker of endocytosis, with and without INF7, were incubated with CV1 cells for 1 hr. After incubation, cells were washed with Ca^2+^/Mg^2+^-free PBS containing 1 mM EDTA to release un-endocytosed liposomes. Cells were then lysed and measured for CF fluorescence that was then normalized to DNA content measured by ethidium bromide (EthBr) fluorescence. The means are not significantly different (*p* = 0.25; *n* = 3).

While artificially and abruptly acidifying the cells led to SR release from endosomal compartments, it was still uncertain whether physiological acidification during the course of endosomal maturation would result in SR release into the cytosol. To investigate this, cells were incubated with liposomes for 1 hr and then washed to remove unendocytosed liposomes. Cells were then incubated for an additional 2 hr in the absence of liposomes and then imaged. After incubation with both types of liposomes, fluorescence in cells was highly heterogeneous in appearance. Importantly, however, the fluorescence was much less intense in cells incubated with INF7-containing liposomes (Fig. 5). Because the liposomes also incorporated CF-PE as a tracer, we were able to demonstrate that initial uptake of INF7^+^ and INF7^-^ liposomes was the same in cells after the 1 hr incubation (*n* = 3, *p* = 0.25; Fig. 6). Since the amount of CF-PE per cell did not differ significantly between cells incubated with either liposomal formulation, the lower intracellular SR fluorescence after liposomal uptake suggests that SR was released from naturally acidifying endosomes by INF7; but once SR entered the cytosol, it became susceptible to cytosolic transport [29] and was rapidly extruded out of the cells.

**Fig 7:**
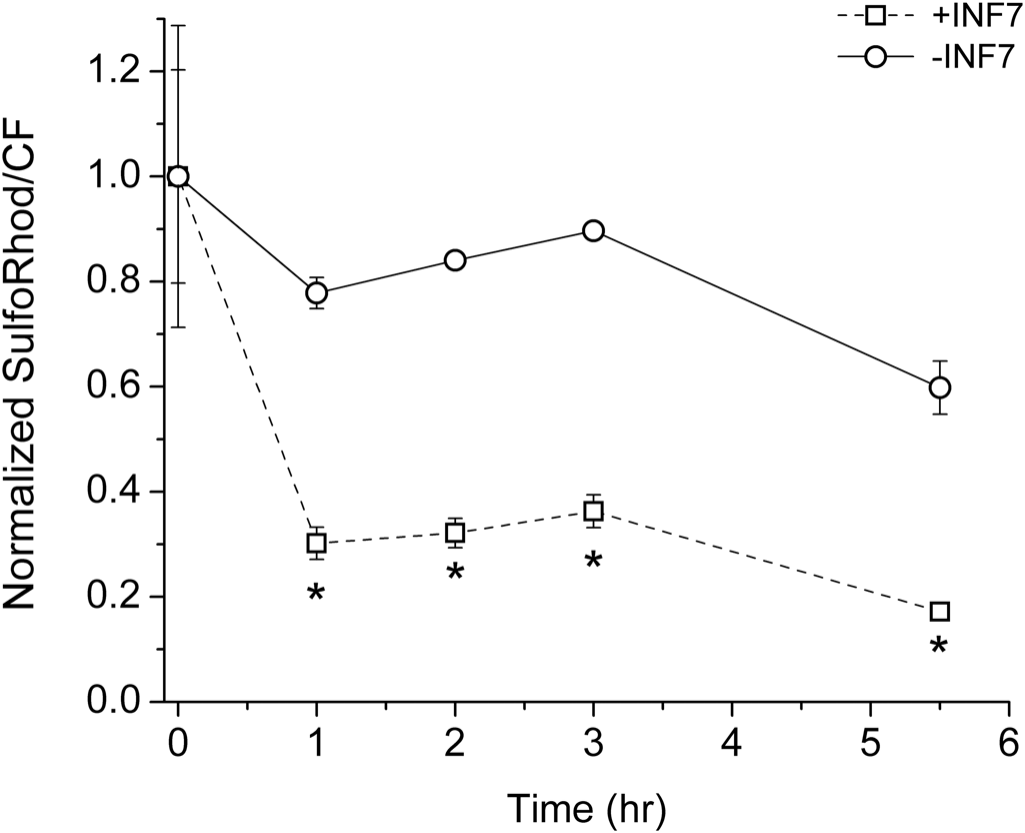
Time course of intracellular SR signal after incubation with liposomes containing or lacking INF7. CV1 cells were incubated with liposomes for 1 hr, washed with Ca^2+^/Mg^2+^-free PBS containing 1 mM EDTA, and allowed to incubate in Ca^2+^/Mg^2+^-containing HBSS without liposomes (time 0). At various time points, cells were trypsinized and lysed (*n* = 3 dishes per time point). Lysates were measured for SR and CF-PE fluorescence and the SR/CF-PE ratio was normalized to the value at time 0. SR fluorescence, relative to CF-PE fluorescence, decays more rapidly in cells receiving INF7-containing liposomes compared to cells receiving liposomes without INF7; this reflects rapid extrusion of SR from the cytosol after SR release from endosomal compartments. Asterisks indicate significance (*p* < 0.05; two-way ANOVA). Error bars indicate the SEM and are not shown when smaller than the plotted symbol.

Intracellular SR retention was also measured by spectrofluorometry. Liposomes with or without INF7 were incubated with cells for 1 hr, washed in divalent-ion-free solution to remove un-endocytosed liposomes. Then at various time points over the subsequent 4 hr, cells were removed from replicate culture dishes (*n* = 3 dishes per time point), lysed and measured for both CF-PE and SR fluorescence (Fig. 7). Intracellular SR fluorescence, relative to CF-PE, decayed more rapidly in cells incubated with INF7-containing liposomes compared to cells incubated with liposomes containing no INF7 (*p* < 0.05). Because cytosolic SR is more efficiently extruded than endosomal SR, the faster loss of intracellular SR fluorescence in the presence of INF7 implies that INF7 activation facilitates SR escape from endosomal compartments.

### Intracellular distribution and retention of rhodamine-labeled dextran during endosomal maturation

**Fig 8:**
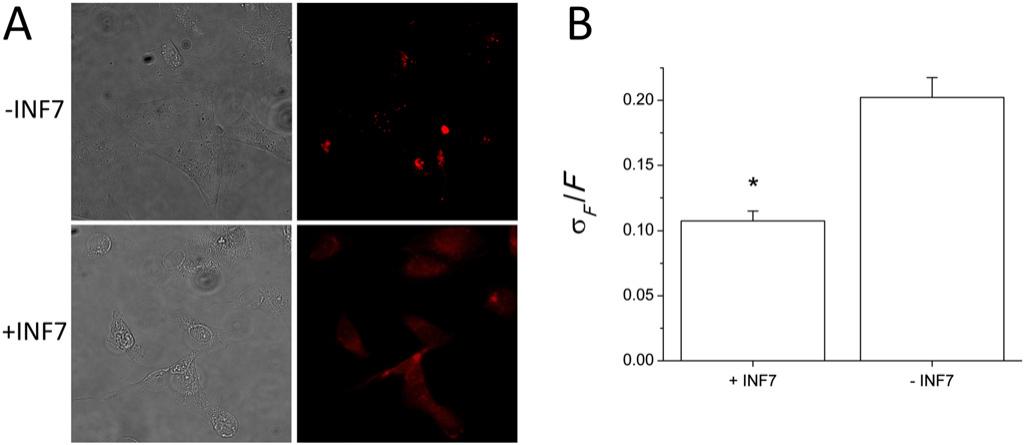
INF7 mediates release of endosomal RD into the cytosol. **A.** Liposomes containing RD with or without INF7 were incubated with CV1 cells for 1 hr, washed to remove un-endocytosed liposomes, and incubated for an additional 2 hr. RD fluorescence appears more evenly distributed throughout the intracellular volume after endosomal release by INF7 (+INF7). In contrast, in cells incubated with INF7-free liposomes, RD is retained endosomally and appears punctate. **B.** The standard deviation of pixel intensity relative to mean pixel intensity (σ_*F*_ */F*) is significantly lower after incubation with INF7 liposomes compared to incubation with INF7-free liposomes (*n* = 5). Error bars indicate SD and asterisk indicates significance (*p* = 6.3 × 10^-6^) determined by *t*-test.

Alternatively, INF7 activation and release of imaging probes can be demonstrated using liposomally-encapsulated rhodamine-conjugated dextran (RD, MW ~4,000). Because cells lack transport mechanisms to extrude nonmetabolizable biopolymers like dextran, fluorescent dextran conjugates are retained in the cytosol for very long periods of time [30]. Liposomes containing RD, either with or without INF7, were incubated with cells for 1 hr. Cells were washed in divalent-cation-free HBSS to remove un-endocytosed liposomes and incubated in fresh HBSS for another 2 hr and then imaged (Fig. 8A). In the case of liposomes containing INF7, because RD is well retained in the cytosol, RD released from endosomes by INF7 appeared uniformly distributed through the intracellular volume (Fig. 8A, +INF panels). In contrast, when liposomes lacking INF7 were used, RD fluorescence remained punctate, reflecting its continued confinement within the endosomal system (Fig. 8A, -INF panels). INF7-mediated release of RD was further confirmed by analyzing the SD of pixel intensity relative to mean pixel intensity (σ_*F*_ */F*) in cellular fluorescence images. Cells receiving INF7-containing liposomes had significantly lower σ_*F*_ */F* than cells that were incubated with INF-free liposomes (*n* = 15; *t*-test *p* = 6.3 × 10^-6^) (Fig. 8B).

## Discussion

We have shown that fluid-phase tracers loaded into the endolysosomal compartment rapidly reappear in the extracellular solution—consistent with known endosomal recycling mechanisms. Poor retention of imaging probes in the endosomal compartments lowers SNR and narrows the time window for cellular imaging. The inability to deliver imaging probes to the nucleo-cytosolic compartment thus limits the application of liposomes for *in vivo* cellular imaging. Therefore, an agent such as an FP, which can facilitate escape of imaging probes from the endolysosomal compartment into the nucleo-cytosolic compartment, may enhance *in vivo* cellular imaging. This study demonstrates that co-encapsulation of the influenza-derived INF7 peptide with rhodamine-type fluorescent tracers in liposomes causes efficient translocation of endosomal rhodamine into the cytosol after endocytosis of the liposomes by CV1 cells (Fig. 1B). We show by fluorescence microscopy that acidification by organic acid acutely activates the fusogenicity of INF7 to enable endosomally-trapped SR to escape rapidly into the cytosol. Moreover, we show that physiological acidification of endosomes in the normal course of endosome maturation also activates INF7 to cause robust release of rhodamine tracers into the cytosol. This is demonstrated by incubating CV1 cells with liposomes encapsulating either SR or RD, with or without INF7. INF7-facilitated escape of SR or RD from the endosome causes cells to show quantitative differences in the fluorescence microscope. When SR, which is a low-MW organic anion, enters the cytosol from endosomes, it is efficiently extruded by cytosolic transporters; this results in weak fluorescence in cells incubated with liposomes encapsulating SR and INF7. In contrast, because RD, being a nonmetabolizable polymer, eludes transporters, it is well retained in the cytosol and appears brighter and uniformly dispersed through the intracellular volume after INF7 activation. Incorporation of a CF-labeled lipid tracer into liposomes permits quantitative monitoring of endocytosis which, in turn, allows quantification of SR and RD relative to the progress of liposome endocytosis. Intracellular fluorescence of endosomally-confined fluid-phase tracers (no INF7) does decay over time as the tracers are lost from the cell through normal endosome recycling [18, 19]. With the inclusion of INF7 in the liposomes however, intracellular SR signal declines more rapidly owing to cytosolic extrusion, while intracellular RD accumulates to greater levels after endosomal release by INF7 because RD, being macromolecular, is not extruded from the cytosol.

Co-encapsulation of INF7 with imaging probes that are resistant to extrusion from the cytosol could greatly improve many *in vivo* cell imaging and cell tracking applications. Both intracellular signal generation and retention are essential to optimal performance of many imaging applications. Liposome technology has been refined over recent years and targeted liposomal delivery of imaging probes *in vivo* can be performed readily. This is especially true for tumors that frequently have aberrant vasculature that permits greater extravasation of liposomes [35]. Circulating liposomes extravasate into interstitial spaces of tumors and can be endocytosed by tumor cells. Furthermore, endocytosis can be selectively targeted through liposomal modification. We have demonstrated that fluorescent probes are better retained intracellularly when their release into the cytosol is mediated by INF7, but *in vivo* fluorescence imaging applications are limited because light in the visible wavelength range use to excite common fluorophores penetrate tissue poorly. However, our group is developing targeted liposomal delivery of imaging probes for *in vivo* electron paramagnetic resonance imaging (EPRI) that could also benefit greatly from the action of INF7.

EPRI is a magnetic resonance modality that is highly promising for *in vivo* cellular imaging. EPRI images the magnetic resonance of unpaired electrons, which is the defining characteristic of free radicals. The body has negligible levels of endogenous free radicals; therefore, EPRI requires the use of exogenous, *stable* free radicals (so-called “spin probes”) as contrast agents. Nitroxides constitute the major class of spin probes used in EPR spectroscopy and imaging. EPRI with nitroxides may be viewed as the magnetic resonance equivalent of optical imaging with fluorophores—i.e., targeting of molecular probes enables us to see features of interest that are otherwise invisible. Nitroxides are thus analogous to fluorophores: they can be used to label cells for visualization or, with appropriate chemical tailoring, they can report on cellular physiology. EPRI, like MRI, uses radiofrequency waves for imaging. Radio waves show excellent tissue penetration, and thus permit imaging of large animals, including humans. This unique combination of attributes gives EPRI the potential to image the vast array of biological phenomena that can be interrogated by fluorescence and the ability to do so in deep tissues *in vivo*.

We have already developed a liposome-based *in vivo* targeting strategy to deliver nitroxides to breast tumor cells that overexpress human epidermal growth factor-2 (HER2) [6]. Analogously to fluorophores, nitroxides exhibit a concentration-dependent self-quenching of their spectroscopic signal. Nitroxides can be encapsulated at self-quenching concentrations in liposomes; such liposomes are spectroscopically “dark”, which minimizes background signal when the liposomes are in the circulation. The liposomes are decorated with anti-HER2 antibody fragments; therefore, when they extravasate into HER2^+^ tumors, they bind to, and are selectively endocytosed by, the HER2^+^ tumor cells. In the endosomal system, disruption of the liposomes causes the release of their nitroxide contents. The consequent dilution relieves quenching to restore “bright” nitroxide signal. This strategy is essentially a targetable, cell-activated contrast-generating mechanism that could be used to “highlight” HER2^+^ lesions for *in vivo* EPRI.

The nitroxides we have delivered to cells by liposomes are ionically charged at physiologic pH and like the fluorophores employed in this study, are retained endosomally and subject to metabolic and endosomal recycling processes. Any improvement in intracellular accumulation would increase signal-to-noise ratio in imaging and potentially the intracellular signal lifetime (the longer the signal persists, the wider the temporal window during which imaging can be performed). The INF7 peptide should also allow release of nitroxides from the endosomes into the cytosol and thus prevent their loss through endosome recycling or potential metabolism. The nitroxide that we have successfully delivered with liposomes is a small molecule bearing three ionic charges at physiological pH, and is susceptible to cytosolic extrusion—it has a cellular retention half-life of *t*_½_ = 79 min [36]. Our earlier work showed that the intracellular retention half-life increases with *Z*_Tot_, the total (not net) number of ionic charges on the nitroxide: log(*t*_½_) = 0.532*Z*_Tot_ + 0.286 [36]. Thus, a nitroxide with a total of 4 ionic charges (e.g., 2 positive and 2 negative) would be expected to have *t*_½_ > 4 hr, which would be very useful for most *in vivo* EPRI experiments. Chemical syntheses of such highly-charged nitroxides are under way.

In summary, liposomes can be targeted to deliver imaging probes into the endosomal system of specific cells *in vivo*. Co-encapsulation of peptides such as INF7 with imaging probes in the liposomes can facilitate transfer of the probe molecules from the endocytic pathway into the cytosol. Rational improvements to the probe molecules would greatly increase the intracellular retention time of the probes and thus widen the temporal window for *in vivo* imaging.

## Supporting Information

S1 Fig Stability of liposomes containing INF7 peptide as function of pH. Liposomes encapsulating 90 mM sulforhodamine B (SR) and 500 liposomes encapsulating 90 mM sulstirred assay buffer in a fluorescence cuvette at 37 liposomes encapsulating integrity causes SR to leak out and be diluted into the assay buffer. The consequent rise of SR fluorescence due to de-quenching gives a sensitive read-out of liposome leakage. Identical measurements were conducted in 9 assay buffer solutions ranging in pH from 3.5 to 8.45. The leakage rate at 180 sec after liposome addition was normalized to the lowest rate observed (which was at pH 8.45), and plotted against pH. The normalized rates (blue open circles) were fit by nonlinear least-squares procedures to a dose-response function (solid red line), whose midpoint occurs at pH = 5.64 integrity causes SR to leak out and be diluted into the assay buffer. The consequent rise of SR fluorescence due extrema.
(PDF)

S1 Video Effect of exogenous acid on endocytosed INF7-containing liposomes. (MP4)

S2 Video Effect of exogenous acid on endocytosed control liposomes lacking INF7. (MP4)
