# Supplementary material for: Co-Encapsulating the Fusogenic Peptide INF7 and Molecular Imaging Probes in Liposomes Increases Intracellular Signal and Probe Retention
Source: PLoS One. 2015 Mar 27;10(3):e0120982. doi: 10.1371/journal.pone.0120982 (PMC4376389; doi:10.1371/journal.pone.0120982)
Supplement: S1 Fig — Liposomes encapsulating 90 mM sulforhodamine B (SR) and 500 liposomes encapsulating 90 mM sulstirred assay buffer in a fluorescence cuvette at 37 liposomes encapsulating integrity causes SR to leak out and be diluted into the assay buffer. The consequent rise of SR fluorescence due to de-quenching gives a sensitive read-out of liposome leakage. Identical measurements were conducted in 9 assay buffer solutions ranging in pH from 3.5 to 8.45. The leakage rate at 180 sec after liposome addition was normalized to the lowest rate observed (which was at pH 8.45), and plotted against pH. The normalized rates (blue open circles) were fit by nonlinear least-squares procedures to a dose-response function (solid red line), whose midpoint occurs at pH = 5.64 integrity causes SR to leak out and be diluted into the assay buffer. The consequent rise of SR fluorescence due extrema. (PDF) [file pone.0120982.s001.pdf]

Figure S1. Stability of liposomes containing INF7 peptide as function of pH.

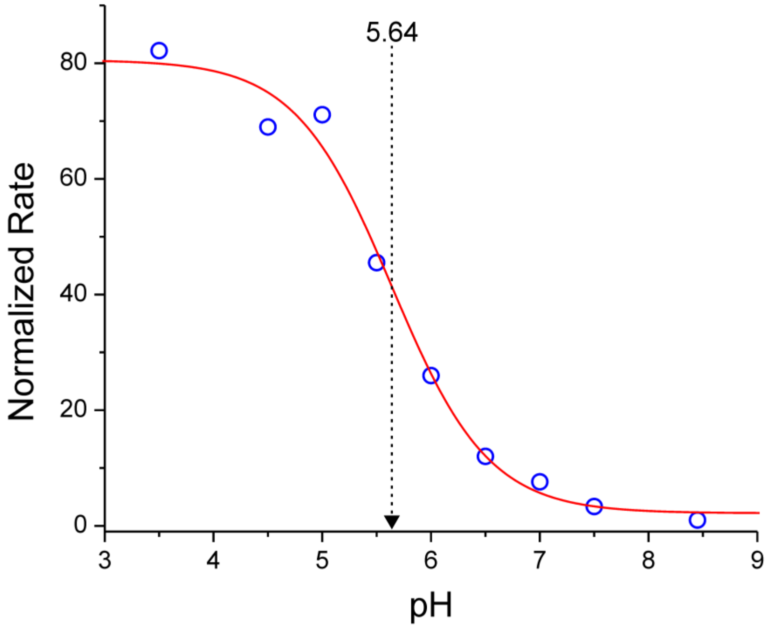

Liposomes encapsulating 90 mM sulforhodamine B (SR) and 500 μg/mL INF7 peptide were added to stirred assay buffer in a fluorescence cuvette at 37 °C. Any loss of liposome integrity causes SR to leak out and be diluted into the assay buffer. The consequent rise of SR fluorescence due to de-quenching gives a sensitive read-out of liposome leakage. Identical measurements were conducted in 9 assay buffer solutions ranging in pH from 3.5 to 8.45. The leakage rate at 180 sec after liposome addition was normalized to the lowest rate observed (which was at pH 8.45), and plotted against pH. The normalized rates (blue open circles) were fit by nonlinear least-squares procedures to a dose-response function (solid red line), whose midpoint occurs at pH = 5.64 ± 0.09, with a Hill coefficient of –0.98 ± 0.18. Measurements were performed in duplicate at every pH except the extrema.
